# Making Digital Health “Solutions” Sustainable in Healthcare Systems: A Practitioner Perspective

**DOI:** 10.3389/fdgth.2022.727421

**Published:** 2022-03-31

**Authors:** Matthew Cripps, Harry Scarbrough

**Affiliations:** ^1^Sustainable Healthcare National Health Service (NHS) England, London, United Kingdom; ^2^Centre for Healthcare Innovation Research, City, University of London, London, United Kingdom

**Keywords:** innovation, adoption, digital health, sustainability, implementation

## Abstract

Digital health solutions have the potential to bring about great improvements in the delivery and quality of services in healthcare systems. In this paper, we draw on the extensive experience of NHS (National Health Service) England to develop a practitioner perspective on the challenges of effectively implementing and sustaining such solutions. We argue that a properly sustainable approach requires a shift in both thinking and practice when it comes to the spread and adoption of such technologies. Our thinking needs to shift from a focus on the technology itself to how we bring about the changes needed to deliver more efficient and effective care for patients. In practical terms, this means focussing on the changes involved to integrate digital health solutions into the delivery of services. In particular, it requires greater attention to the motivations, constraints and specific contexts that influence users and patients. The technical expertise of innovators therefore needs to be complemented by other forms of insight into change processes, including clinical and behavioral insight, process engineering and knowledge management. In this paper, we show how these different pillars of the NHS Sustainable Healthcare approach help to ensure the effective implementation and use of digital solutions. We draw out the implications of this approach for policy-makers in healthcare systems, highlighting the need to give greater attention and resources to the downstream challenges of implementing digital health solutions.

## Introduction

The emergence of a new wave of healthcare innovations based on digital technology has raised great hopes and expectations around the transformation of healthcare systems to offer more effective, efficient and personalized services in the future. A number of reports have highlighted the potential benefits to be gained from different forms of digital health technology (1, 2). In this article, we build on experience gained from the NHS in England to argue that expectations based on the potential of these digital technologies need to be balanced by greater recognition of, and investment in, the forces that enable them to be successfully and sustainably applied in practice (3, 4). We should not expect these new technologies to bring about sustainable improvements in healthcare in their own right. In fact, the assumption that digital technology is *the solution* to healthcare problems is a major barrier to bringing about the sustainable change that we need (5).

The evidence for the limitations of a technology-driven approach in healthcare is extensive (6). Many of the past failings of EHR (Electronic Health Record) systems, for example, were attributed to the lack of user-centered design (6). Such systems met the needs of the hospital administrators, but were not seen as meeting the needs of clinicians (7). This example highlights two important limitations of the technology-driven approach. First, the focus is on technology and not on the transformation of practices or processes. Wachter, for example, argue that we need to distinguish between such “technical change” and the “adaptive” approach to change required in healthcare (8). As a Nuffield Trust report on the spread of digital technology in the UK healthcare system observed: “Where technological interventions have failed, technology has simply been layered on top of existing structures and work patterns, creating additional workload for health care professionals” (1). Second, the design of systems fails to take account of the user. As this same report notes; “staff are too often seen as “passive recipients” of new technology and not involved in the development of systems architecture or user interfaces”. In short, as another recent report puts it; “The barriers to uptake are often the patient or user and badly-designed and implemented technology” (7).

We argue instead that a properly sustainable approach requires both a shift in our thinking and in our practice when it comes to the spread and adoption of digital health technologies. Our thinking needs to shift from a focus on the technology itself to how we bring about the changes needed to deliver more efficient and effective care for patients. In practice, this involves being much more focussed on how digital technologies will be integrated and applied in services, and the motivations, constraints and specific contexts that influence those applying them and benefitting from them (9). Digital technology is viewed as one strand in a process of change, and the change itself is seen as nested within a wider policy environment and specific contexts which may also need to be changed or adapted.

## NHS Sustainable Healthcare Approach

**Figure 1:**
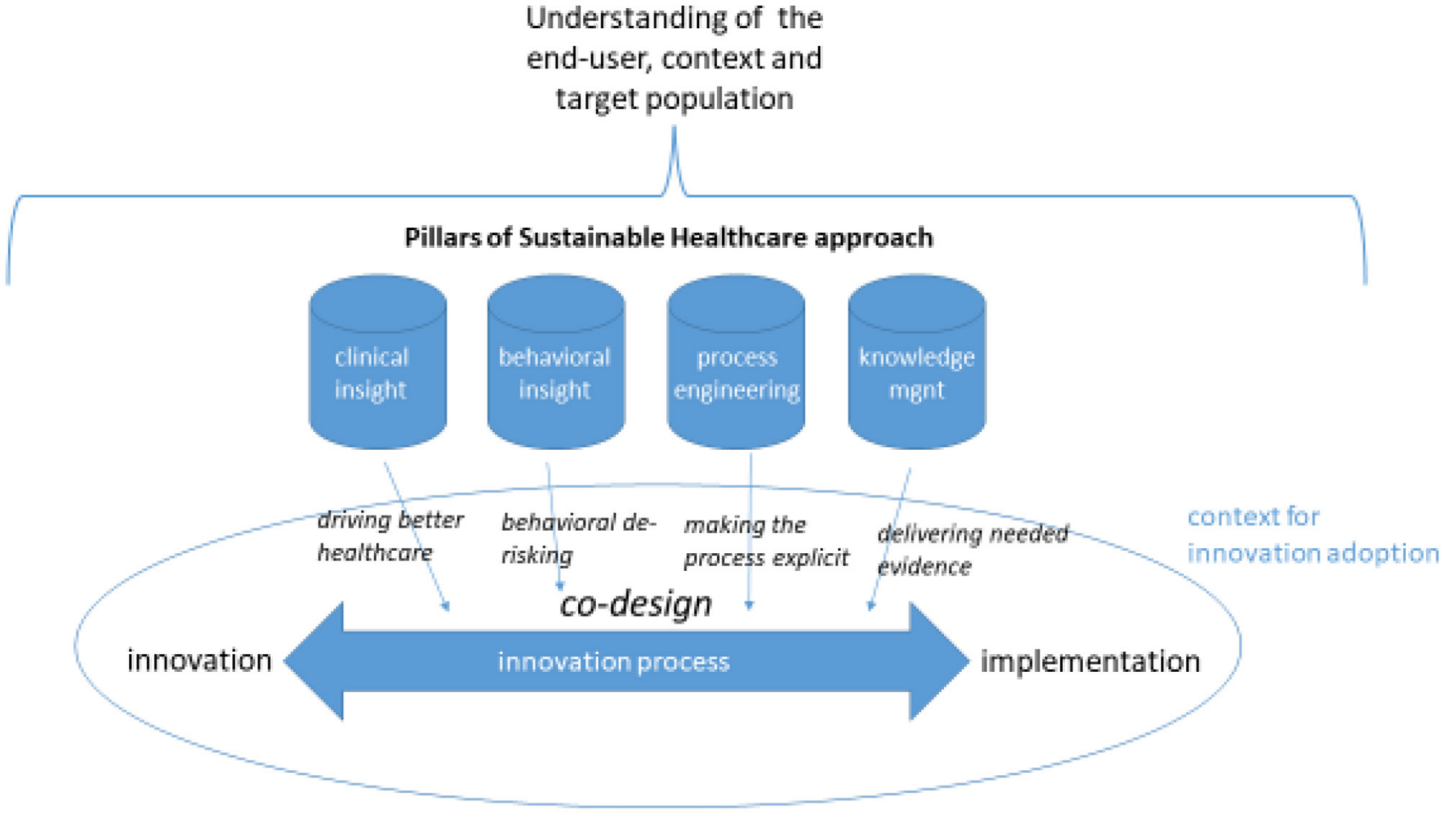
NHS sustainable healthcare approach to digital innovation.

At NHS England, this shift in thinking and practice is exemplified by the work of the Sustainable Healthcare team. This team has developed a programmatic approach through which new digital applications are integrated into a wider process of change which begins in the early stages of development and extends through to the long run sustainable implementation of an innovation or intervention. Making the adoption of digital health technologies sustainable involves understanding and addressing this complexity in the change process (3). As outlined in Figure 1, this approach rejects the traditional linear model of the innovation process in favor of an interactive model where implementation is not an afterthought but a primary focus of co-design efforts. The process of change applied by the NHS Sustainable Healthcare team does this by injecting four pillars of insight.

### Pillars of Insight

The first of these pillars is *clinical insight*. In other words, not losing sight of the ultimate purpose of the innovation. Whatever form of change is being introduced in the NHS, even if it is a logistics or catering project, the ultimate purpose is to support delivery of better healthcare to the population. Any change process within the NHS needs to take account of this purpose. Changes which do not serve this purpose, or which create barriers to it, will not be sustained over the longer term.

The second pillar is *behavioral insight*. This draws on a range of disciplines including psychology, sociology and data sciences to understand how people might respond to new services or technologies. The aim is to “nudge” people so that evidence-based change is positively received by framing it appropriately in communications around the project. Taking the responses of end users into account from the early stages of design in this way allows for “behavioral derisking” to pre-empt implementation problems.

The third pillar is *process engineering*. The aim here is to make the process of applying the new technology or service as explicit, simple and comprehensible as possible. In the dialogue between the team, adopters and users, flowcharts are developed that clearly map out the steps involved, and their sequencing. People can be confident that if they follow the process steps, they will get the right outcomes more often. Making the process explicit helps, whether it is self-managed care or the running of an outpatient department. It also helps to guide changes in the context to make sure that a new technology or service can be delivered effectively.

Finally, *knowledge management* provides valuable insight on the evidence underpinning the need for change, and how best to deliver it. It is important that efforts are directed toward the right kind of change so that the ultimate purpose is achieved. This is something that needs to be addressed throughout the change process, so that whatever direction it takes and whatever outcomes are achieved, they are demonstrably based on the best available evidence.

### Understanding of End Users

As highlighted by Figure 1, these pillars build from the outset on an understanding of the likely responses and motivations of the “end users” of the technology, including public and frontline staff users, and the context in which they will be using it. Effective change processes need to incorporate perspectives from all such user groups (10). Frontline staff groups, for example, are doing a lot more than use the technology—they are making it an active part of their service delivery and the treatment of patients. This requires intensive communication to ensure that staff understand, own, and are motivated to bring about the changes that are in needed in their practices and ways of working.

The adopters and users of technology are too often an afterthought for developers who are more concerned with the functionality of their kit. But this risks resistance or poor take up of new digital tools, not by intention but by failing to consider the users' needs and responses to what is being proposed (3, 11). The Sustainable Healthcare team's approach is always to identify the target audience or population early, and engage with them or their representatives to understand the “end user”, in particular the behavioral barriers and drivers to them adapting to and adopting the innovation or new behavior. This also allows for co-design and ensures that the system's efforts to support mobilization are informed by what the end user needs to be enabled. Acceptance and sustained use of an intervention or innovation is much greater if people feel that it has been designed *with them*, not dropped *on them*. This is as much the case for a new digital health application as for any other change (10).

The need for this approach is reinforced where the user population is more diverse and less homogeneous. Neurological diversity may be an important challenge, for example; people with autism, dyspraxia, and dyslexia might be unintentionally prevented from interacting with a digital app if it is not designed to account for their needs. Similar challenges apply to different levels of digital literacy, access to the internet, and so on (12).

### The Importance of Context

The responses of adopters and users are also shaped by the context in which they operate, which tends to buttress the status quo against change (9). Failure to anticipate these contextual influences can quickly undermine even the most beneficial of innovations. One example here is the powerful influence of financial and incentive systems. To cite a recent instance of this, the COVID-19 pandemic prompted the widespread introduction of virtual consultations in place of outpatient attendance at hospitals in the NHS. This innovation has arguably produced some positive benefits for patients in terms of easier access to services, but its spread and sustainability may ultimately be dependent on its financial consequences. If the effect of virtual consultations were to reduce the number of more financially lucrative in-person consultations with hospital-based consultants, this may create a perverse incentive for hospitals to back away from the use of this innovation.

To further underline the importance of context, the greater use of virtual consultation implies concomitant changes on the primary care side, including the need for greater resources to support primary and community care, and the need to create a new group of advanced care practitioners to take some of the increased burden away from GPs. All of these ripple effects from the introduction of new virtual consultation technologies mean that the changes involved will not be sustainable if the context is not also changed to accommodate them.

This change approach is based on a set of principles and is applied flexibly rather than dogmatically. It works best when the NHS Sustainable Healthcare team are involved from the earliest stage, but they are sometimes co-opted later in the process to help overcome barriers to adoption and use. This often involves bringing a sharper focus to the responses of end users, seeing how change cascades through different levels; asking, for example, what are the barriers to senior leaders or clinical champions deciding to do this, and then what about the front line staff, who may be being asked to adopt new changes every day? Beyond that, we need to ask how do the frontline engage with the wider population, and how do comms and media help persuade that population? At each level, and for each group, it is important to ask; what are the barriers, what are the drivers?

This approach has produced excellent outcomes in a number of cases. But it has to be applied in a reflective and adaptable way. One technique used by the team to ensure this is called “pre mortem planning”. The aim of this technique is to encourage team members to tease out prospectively the kind of problems or unintended effects in a change process which would normally only be picked up retrospectively, by virtue of the effort to leverage change being unsuccessful, or less successful than aimed for. It permits a project team to imagine, pre-emptively, that their planned approach does not work and ask the question; how did this project fail? This technique frees team members up to take a more critical stance toward their change plans, their design prototypes etc., and thus avoids any kind of groupthink creeping into their deliberations.

## Discussion

The approach to change described here represents a response to the specific challenges of implementing and sustaining digital health innovations in NHS England. At the same time, many of its features echo a body of practitioner and policy-maker experience with digital innovation which is emerging globally. For example, UNICEF has long emphasized “human-centred design” in its work^1^, and many international development organizations have benefitted from applying the ideas and resources created by the Principles of Digital Development organization^2^, including the formulation of principles such as “design for scale” and “build for sustainability”. Within the digital health field specifically, recognition of the contribution which digital innovations can make to healthcare globally, including in LMIC countries, is increasingly matched by an understanding of the challenges which need to be overcome in scaling and sustaining such innovations. Studies have highlighted, for example, the importance of policy-level barriers which may reinforce other challenges such as the mobilization of evidence for innovation (13). Factors seen as critical to overcoming these challenges include gaining input from end-users from the outset and an understanding of the ecosystem within which the innovation is being deployed (14). As evidence mounts on the impact of different digital innovation programmes globally, these key findings are contributing to a more holistic appreciation of the scope of the wider changes involved in making them successful and sustainable, including “systems integration” to ensure implementation support, and ultimately the creation of a supportive digital health ecosystem (15).

While the Sustainable Healthcare team's approach is focussed within the NHS at the organization and individual rather than ecosystem level, this work from the global health field does raise some important questions about the sustainability of innovative digital health solutions once adopted. In this respect, the Sustainable Healthcare attention to context reinforces the finding from these other studies that a supportive context is vital to the scalability and sustainability of digital innovations (16). One question which arises, however, is whether this focus on context requires the Sustainable Healthcare team to apply a different approach to digital vs. non-digital innovations. Since their approach is attuned to behavioral and contextual features more than to the characteristic features of the innovation, it is clearly more concerned with how an innovation *lands* in a specific context. To that extent, it may be viewed as being agnostic toward the particular form of technology being deployed within a process of change. However, it is important recognize that there are also distinctive features of digital technology which in themselves may serve to raise or lower the barriers to adoption or use. This particularly applies where digital technology is being introduced into an analog world; that is, an environment where policies, infrastructures and practices have not been adapted to the potential of digital health (6). One emerging concern in the adoption of new AI technologies in healthcare, for example, is that they may fall foul of elaborate information governance requirements that make it difficult to access patient data effectively (17).

A further issue arising from the Sustainable Healthcare team's experience with this approach to change has to do with the value of their public health and end-user perspective on the adoption of innovations. Much research and policy interest in this area has focussed on what has been termed the “front end” of innovation; that is the development of new technologies and processes aimed at “early adopter” groups of individuals and organizations. Greenhalgh, for example, comments in a recent report that: “Planners and policymakers have often been overly focused on technologies and distracted by simplistic models and metaphors of technology adoption by individuals” (7).

Viewing innovation adoption from the perspective of end users and the wider public, however, involves a greater focus on how new tools and solutions are used, and how they can be spread to the widest possible extent. This may involve placing a much greater emphasis on the usability and accessibility of digital health solutions—having the right infrastructures and skills in place, for example—and much less on the cutting-edge properties of the solution itself (8). And where the digital solution is highly innovative, this perspective suggests a more intense focus on how it can be spread and used effectively, especially since it is often challenging to bridge the gap between early and later adopters of an innovation (18); something which is observable in the many pilot implementations which never achieve widespread take up (19).

This shift in perspective away from the front end of innovation seems to be an important requirement for the sustainable adoption of digital health solutions in healthcare. But, its implications are not only practical. It also raises important questions about healthcare policy and whether it is sufficiently attuned to the challenges of spreading digital health solutions in a sustainable way. We need to remember that many innovative technologies and treatments have been introduced into healthcare without ever being spread widely or used effectively, despite ample evidence of their benefits to patients (20). Worryingly, policy-makers attracted to the progressive imagery of new digital technologies may be more likely to allocate resources to their front end development than to the more complex downstream challenges of putting them into practice. As a recent UK report puts it; “Government innovation policies seem often to imply a relatively simple hand-over from innovators to technology-savvy commissioners and purchasers, who in turn pass the wizardry onto willing patients, users, and consumers” (6). This may result in a slow and uneven spread of digital health solutions which not only deprives patients of their clinical benefits, but also increases health inequalities amongst the wider population (21).

## Data Availability Statement

The original contributions presented in the study are included in the article/supplementary material, further inquiries can be directed to the corresponding author/s.

## Author Contributions

MC provided the practitioner perspective and practical examples for the paper. HS provided input from academic research and literature. HS and MC wrote the paper. Both authors contributed to the article and approved the submitted version.

## Conflict of Interest

The authors declare that the research was conducted in the absence of any commercial or financial relationships that could be construed as a potential conflict of interest.

## Publisher's Note

All claims expressed in this article are solely those of the authors and do not necessarily represent those of their affiliated organizations, or those of the publisher, the editors and the reviewers. Any product that may be evaluated in this article, or claim that may be made by its manufacturer, is not guaranteed or endorsed by the publisher.

## Footnotes

^1^https://www.unicef.org/innovation/hcd.

^2^ https://digitalprinciples.org
